# The Physician’s Visiting List for 1893

**Published:** 1892-12

**Authors:** 


THE PHYSICIAN'S VISITING LIST for 1893. (Lindsay & Blakiston's.) Philadelphia: P. Blakiston, Son & Co., 1012 Walnut Street.

This is the forty-second year of the publication of this well-known visiting list. It measures $6\frac{1}{8}$x$3\frac{7}{8}$ inches, and is the smallest and lightest visiting list published. Among its valuable features may be mentioned the relation of the French metric system to English weights and measures, tables of dosage, etc., poisons and their antidotes, examination of urine, a table of the forms of Bright's disease showing the differences of the various forms; diagnoses of the simpler diseases of the eye; tables of the eruptive fevers, showing differential diagnosis, and other tables that are used daily by the physician in his rounds. It is bound in morocco, furnished with tuck, pocket, and pencil. The price is, for 25 patients a week, $1.00; 50 patients, $1.25; 75 patients, $1.50; 100 patients, $2.00. This visiting list has been used by the editor of this journal for a number of years.

